# *Strain* do Átrio Esquerdo pela Técnica de *Speckle-Tracking* : Contribuição para a Avaliação da Função Diastólica em Pacientes Pediátricos com Doença Renal Crônica

**DOI:** 10.36660/abc.20230131

**Published:** 2024-04-12

**Authors:** Flora Maciel Penachio, Maria de Fátima Rodrigues Diniz, Rosana Sbruzzi Prado Laurino, Andreia Watanabe, Karen Saori Shiraishi Sawamura, Alessandro Cavalcanti Lianza, Carolina Rocha Brito Menezes, Isabela de Sousa Lobo Silva, Gabriela Nunes Leal

**Affiliations:** 1 Universidade de São Paulo Faculdade de Medicina São Paulo SP Brasil Universidade de São Paulo – Faculdade de Medicina, São Paulo, SP – Brasil; 2 Universidade de São Paulo Instituto da Criança São Paulo SP Brasil Universidade de São Paulo Instituto da Criança, São Paulo, SP – Brasil; 3 Universidade de São Paulo Hospital das Clínicas São Paulo SP Brasil Universidade de São Paulo Hospital das Clínicas, São Paulo, SP – Brasil; 4 Hospital do Coração São Paulo SP Brasil Hospital do Coração, São Paulo, SP – Brasil; 5 Hospital Israelita Albert Einstein São Paulo SP Brasil Hospital Israelita Albert Einstein, São Paulo, SP – Brasil; 6 Hospital Sírio Libanês São Paulo SP Brasil Hospital Sírio Libanês, São Paulo, SP – Brasil

**Keywords:** Átrios do Coração, Rim, Ecocardiograma, Criança

## Abstract

**Fundamento:**

As complicações cardiovasculares são a principal causa de morte em pacientes pediátricos com doença renal crônica (DRC). A avaliação ecocardiográfica da função diastólica na DRC tem se limitado à avaliação espectral por Doppler espectral e por Doppler tecidual, técnicas sabidamente menos confiáveis na pediatria. O *strain* do átrio esquerdo (AE) pela técnica do *speckle tracking* bidimensional (2DST) foi recentemente confirmada como uma medida robusta da função diastólica.

**Objetivos:**

Investigar o papel do *strain* do AE na avaliação da função diastólica de crianças em diferentes estágios da DRC.

**Métodos:**

De fevereiro de 2019 a julho de 2022, 55 pacientes com DRC sem sintomas cardiovasculares e 55 controles foram avaliados por ecocardiografia convencional e por ecocardiografia com 2DST. O nível de significância adotado foi de 5% (p < 0,05).

**Resultados:**

Pacientes e controles tinham idade similares [9,78 (0,89 – 17,54) vs. 10,72 (1,03 –18,44) anos; p = 0,41] e sexo (36M:19F vs. 34M:21F; p = 0,84) similares. Havia 25 pacientes não dialíticos e 30 pacientes dialíticos. A fração de ejeção do ventrículo esquerdo foi ≥ 55% em todos. Em comparação aos controles, os pacientes com DRC apresentaram *strain* de reservatório mais baixo (48,22±10,62% vs. 58,52±10,70%) e índice de rigidez do AE mais alto [0,14 (0,08–0,48)%^-1^ vs. 0,11 (0,06–0,23) %^-1^]; p<0,0001. A hipertrofia ventricular esquerda associou-se com um *strain* de reservatório mais baixo (42,05±8,74% vs. 52,99±9,52%), e valores mais altos de índice de rigidez [0,23 (0,11 – 0,48)%^-1^ vs. 0,13 (0,08–0,23) %^-1^ e de índice de enchimento do AE (2,39±0,63 cm/s x %^-1^ vs. 1,74±0,47 cm/s x %^-1^; p<0,0001). Hipertensão não controlada associou-se com *strain* de reservatório do AE mais baixo (41,9±10,6% vs. 50,6±9,7; p=0,005).

**Conclusão:**

O *strain* do AE mostrou-se uma ferramenta útil na avaliação de pacientes pediátricos com DRC e associado com fatores de risco cardiovasculares conhecidos.

**Figure:**
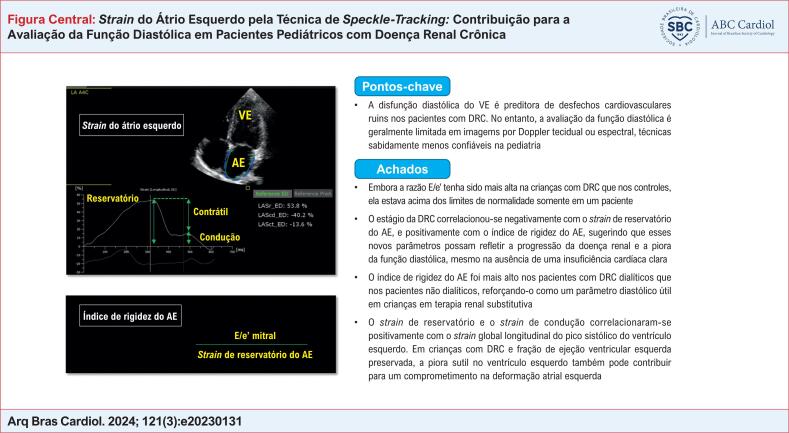


## Introdução

As complicações cardiovasculares são a principal causa de mortalidade em crianças e em adolescentes com doença renal crônica (DRC), sendo responsáveis por até 30% de mortes nessa população.^1^ Tais dados contrastam com os da população pediátrica geral, em que a mortalidade por doença cardiovascular é muito baixa, menos de 3%. Apesar dos avanços na terapia renal substitutiva, as taxas de mortalidade por doenças cardiovasculares em pacientes pediátricos com DRC não mudaram significativamente nas últimas décadas.^2^

O remodelamento do miocárdio na DRC é tradicionalmente considerado uma adaptação fisiológica para diminuir o estresse na parede ventricular em resposta à sobrecarga de volume e hipertensão. No entanto, há vários outros fatores que contribuem para remodelamento e disfunção diastólica do ventrículo esquerdo (VE), tais como toxinas urêmicas, anemia, FGF23, hiperfosfatemia, hiperparatireoidismo e fibrose induzida por estresse oxidativo pela ativação do sistema renina-angiotensina-aldosterona.^3^

A disfunção diastólica do VE é comum em pacientes com DRC e tem sido associada a desfechos cardiovasculares ruins.^4^ Contudo, a maior parte da literatura existente sobre a avaliação da função diastólica em crianças com DRC limita-se a exames de Doppler espectral e Doppler tecidual, técnicas que sabidamente são menos confiáveis em pediatria. Um estudo recente de Dragulescu et al.^5^ demonstrou que parâmetros diastólicos derivados de estudos com adultos não são adequados ou suficientemente discriminatórios na infância.^5^ Além disso, a ampla gama de valores normais de referência em pediatria permite o diagnóstico de disfunção diastólica somente em uma pequena proporção de pacientes.^6^

Dada sua relação dinâmica com a função do VE, o átrio esquerdo (AE) reflete mudanças nas pressões de enchimento, tornando-o um marcador sensível de disfunção diastólica.^7^ Recentemente, a análise da deformação ( *strain* ) do AE por ecocardiografia bidimensional com *speckle tracking* (2DST) confirmou-se como uma medida robusta da função atrial esquerda em diferentes cenários clínicos.^8^ O AE exerce um papel crítico na manutenção do enchimento do VE, atuando como um reservatório para o fluxo venoso pulmonar durante a sístole ventricular esquerda, um condutor do fluxo sanguíneo para o VE durante a diástole precoce e como uma bomba de propulsão durante a diástole tardia.^8^ Alterações no *strain* do reservatório do AE precedem mudanças no volume do AE, favorecendo seu uso para detectar disfunção diastólica subclínica.^9^ O índice de rigidez do AE, calculado como a razão entre o E/e’ e o *strain* do reservatório do AE, foi capaz de diferenciar as crianças com cardiomiopatia dos controles saudáveis, com boa acurácia.^10^ O índice de enchimento do AE, calculado como a razão entre o E mitral e o *strain* do reservatório do AE, mostrou melhor desempenho diagnóstico para identificar um aumento na pressão de enchimento atrial esquerdo que o E/e’. Além disso, um estudo recente demonstrou que o *strain* do reservatório do AE foi um preditor independente de morte cardiovascular e eventos adversos em pacientes adultos com DRC.^11 , 12^

Uma vez que a avaliação da função diastólica por ecocardiograma continua um desafio na pediatria, com ausência de parâmetros padrão-ouro, a incorporação de novas modalidades como a análise da deformação do AE pode ser útil. Com base nessas considerações, o presente estudo teve como objetivo investigar o papel do *strain* do AE na avaliação da função diastólica em crianças e adolescentes em diferentes estágios de DRC.

## Métodos

### Delineamento e população do estudo

Entre fevereiro de 2019 e julho de 2022, 55 pacientes consecutivos com DRC foram recrutados durante suas visitas rotineiras ao ambulatório da nossa Unidade de Nefrologia Pediátrica. Nenhum paciente apresentou sintomas de insuficiência cardíaca (classe I pela *New York Heart Association* ), e cardiopatias congênitas haviam sido descartadas por avaliações ecocardiográficas anteriores. Critérios de exclusão incluíram má qualidade da imagem e recusa do paciente em participar no estudo. O grupo controle foi composto de 55 voluntários sadios oriundos de clínicas da atenção primária, sem história de doença cardiovascular e com ecocardiogramas normais. O comitê de ética da nossa instituição aprovou este estudo transversal, e todos os participantes e representantes legais assinaram um termo de consentimento.

Dados clínicos e demográficos foram obtidos dos prontuários médicos dos pacientes, que foram cuidadosamente revisados pelo médico atendente no dia do ecocardiograma. Dados demográficos incluíram idade, sexo, peso seco, altura, e Área de Superfície Corporal (ASC) calculada pela fórmula de Haycock.^13^ Os dados clínicos incluíram etiologia da DRC, presença, tipo e duração da diálise; presença de hipertensão; medicamentos para doença cardiovascular; hematócrito;^14^ e níveis de fósforo e hormônio da paratireoide (PTH).^15^ De acordo com as recomendações da força tarefa, a hipertensão foi definida pela presença de pressão arterial sistólica e/ou diastólica acima do percentil 95 para idade, sexo e altura da criança.^16^ A classificação da DRC foi baseada na Taxa de Filtração Glomerular (TFG), estimada pela fórmula de Schwartz: estágio I (TFG > 90 mL/min/1,73 m^2^); estágio II (TFG entre 60 e 89 mL/min/1,73 m^2^); estágio III (TFG entre 30 e 59 mL/min/1,73 m^2^); estágio IV (TFG entre 15 e 29 mL/min/1,73 m^2^) e estágio V (TFG < 15 mL/min/1,73 m^2^).^17^

Ecocardiograma 2DST e padrão foram obtidos pelo mesmo cardiologista pediátrico, cego quanto aos prontuários médicos. O examinador, porém, tinha conhecimento de quem era paciente e de quem era controle. Pacientes em diálise foram avaliados entre quatro e seis horas após a última sessão.

### Ecocardiograma padrão

A ecocardiografia transtorácica convencional foi realizada de acordo com as recomendações da *American Society of Echocardiography* (ASE) e incluiu a avaliação em “modo M”, imagem bidimensional, convencional, e Doppler tecidual no anel mitral septal e lateral.^18^ O equipamento usado foi um Philips Affiniti 70 (Andover, MA 01810 USA), com transdutores de multifrequência (S 5-1 e S 8-3 MHz). As dimensões da câmara cardíaca foram obtidas no modo bidimensional, e a Fração de Ejeção do Ventrículo Esquerdo (FEVE) foi calculada pelo método de Simpson. Os diâmetros das câmeras cardíacas, bem como a espessura da parede septal e posterior, foram expressos como valores de z-score.^19^ A massa do VE foi estimada usando a fórmula de Devereaux de acordo com a convenção de Penn e indexada pela altura elevada a uma potência exponencial de 2,7.^18^ O percentil do Índice de Massa Ventricular Esquerda (IMVE) foi calculado para cada paciente, de acordo com os intervalos de referência específicos para idade, propostos por Khoury et al.^20^ A Espessura Relativa da Parede (ERP) do VE foi calculada como a soma da espessura da parede septal e posterior dividida pelo diâmetro diastólico do VE (valor normal ≤ 0,42). A geometria do VE foi classificada como remodelamento concêntrico (ERP anormal e IMVE normal), hipertrofia concêntrica (ERP e IMVE anormais) e hipertrofia excêntrica do VE (IMVE anormal e ERP normal).^20^

A avaliação da função diastólica incluiu medidas de Doppler tecidual e de Doppler convencional – velocidade E mitral, velocidade A, razão E/A, e razão E/e’, sendo e’ a média dos valores obtidos por Doppler tecidual no anel septal e lateral. O volume atrial esquerdo foi estimado usando o método biplanar área-comprimento ao final da sístole ventricular, e os valores foram indexados à ASC.^18^

### Ecocardiograma 2DST

**Figura 1:**
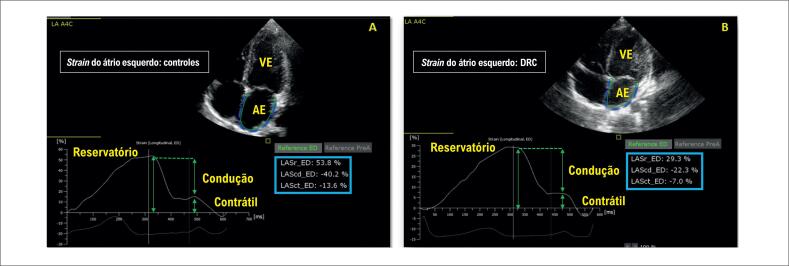
Componentes do *strain* do átrio esquerdo. A: Controle; B: Doença Renal Crônica (DRC); LASr: *strain* de reservatório; LAScd: *strain* de condução; LASct: *strain* contrátil; todos os componentes estão reduzidos na DRC; VE: ventrículo esquerdo; AE: átrio esquerdo.

Imagens em *cine loop* bidimensionais do AE foram obtidas do corte apical de quatro câmaras e armazenadas digitalmente para análise offline do strain por speckel-tracking, usando um programa específico (Q Lab 15, Philips Medical Systems). A taxa de quadros ( *frame rate* ) foi de 80 a 90 quadros/segundo para assegurar um rastreamento ( *speckle-tracking* ) adequado. Tomou-se cuidado para se obter as imagens apicais reais, evitando-se o encurtamento da imagem ( *foreshortening* ). Em segmentos com rastreamento insuficiente, aplicou-se um reajuste manual da borda endocárdica para otimizar sua qualidade. O traçado do AE para determinar o *strain* terminou 0,5cm acima da junção atrioventricular, para evitar a influência da movimentação anular mitral.^21^ O início da onda R no eletrocardiograma foi usado como ponto zero de referência da análise do *strain* . O *strain* do reservatório do AE foi definido como o pico de *strain* sistólico, um pouco antes da abertura da válvula mitral. Isso foi seguido de um platô e um segundo pico tardio no início da onda P indicando o *strain* de contração. O *strain* do conduto foi calculado como a diferença entre o strain de reservatório e o de contração^21^ ( Figura 1 ). O índice de rigidez do AE foi calculado como a razão entre E/e’ e *strain* de reservatório do AE,^10^ e o índice de enchimento do AE como a razão entre o E mitral e o *strain* de reservatório do AE.^11^

Para avaliar a deformação sistólica longitudinal global do VE, imagens bidimensionais em cine-loop dos cortes apical de duas, três e quatro câmaras foram adquiridas e digitalmente armazenadas para análise. Foram escolhidos um ângulo de 30-60° para escaneamento por setor e *frame rates* de 80–90 Hz. O traçado endocárdico foi automaticamente gerado pelo algoritmo computacional (Q Lab 15, Philips Medical Systems) e ajustado manualmente quando necessário. O *strain* longitudinal global do pico sistólico do VE foi calculado, representando valores médios dos 17 segmentos ventriculares analisados nos três cortes.^22^

### Análise estatística

As análises estatísticas foram realizadas usando o programa R com o ambiente de desenvolvimento integrado R Studio (Versão 4.1.0, RStudio, Inc).

Os dados categóricos foram apresentados como frequência relativa e absoluta, e os dados contínuos como média ± Desvio Padrão (DP) ou mediana (intervalo interquartil). O teste de Kolmogorov-Smirnov foi usado para verificar a normalidade da distribuição dos dados. O teste t de Student não pareado foi usado para avaliar dados contínuos com distribuição normal e o teste de Mann-Whitney para avaliar os dados contínuos sem distribuição normal. O teste one-way ANOVA foi usado para comparar mais de dois grupos quanto às variáveis com distribuição normal; o teste de Kruskal-Wallis foi escolhido para variáveis sem distribuição normal. Em ambas as situações, comparações múltiplas foram conduzidas no teste post-hoc, aplicando-se o procedimento de Bonferroni.

O teste do qui-quadrado foi usado para comparar os dados categóricos. O coeficiente de correlação de Spearman foi usado para investigar a relação entre os parâmetros obtidos por ecocardiografia com 2DST e pela técnica padrão. O nível de significância foi estabelecido em 5% (p<0,05).

A variabilidade intraobservador e entre observadores quanto às medidas de 2DST foi testada. O primeiro examinador repetiu a análise de 20 pacientes com DRC e 20 controles sadios selecionados aleatoriamente, três meses após a aquisição das imagens. A randomização dos participantes consistiu em sortear um número (seus números de registro) de uma caixa.

Um segundo observador, que desconhecia os resultados anteriores, também realizou a análise *offline* dos mesmos indivíduos.

A variabilidade intraobservador e entre observadores nas medidas de *strain* foi avaliada usando o Coeficiente de Correlação Intraclasse (CCI). Um CCI maior que 0,8 foi considerado como uma boa correlação.

## Resultados

### Dados demográficos e clínicos

Pacientes com DRC e controles eram similares quanto à idade [9,78 (0,89 – 17,54) anos vs. 10,72 (1,03 – 18,44) anos; p=0,41] e distribuição de sexo (36M:19F vs. 34M:21F; p=0,84). Conforme o esperado, peso seco, altura e ASC eram significativamente menores nos pacientes com DRC ( Tabela 1 ).

**Tabela 1:** Pacientes com doença renal crônica (DRC) vs. controles: dados demográficos, parâmetros da ecocardiografia convencional e por ecocardiografia bidimensional com speckle tracking (2DST)

Dados demográficos		DRC (n=55)	Controles (n=55)	Valor p
Idade (anos)		9,5 ± 4,9	10,4 ± 5	0,3878
Sex (male)		36 (65,45%)	34 (61,81%)	0,8429
Peso seco (kg)		25 (5 – 100)	43 (10 – 84)	0,0009
Altura (m)		1,29 (0,66 – 1,85)	1,45 (0,74 – 1,84)	0,0053
ASC (m^2^)		0,97 ± 0,42	1,25 ± 0,44	**0,0009**
Frequência cardíaca (bpm)		85 ± 14	90 ± 25	**0,1900**
**Parâmetros da ecocardiografia convencional**				
	DDVD (z-score)	-0,41 ± 1,3	-0,22 ± 1,0	0,39
	DDVE (z-score)	-0,31 ± 1,3	-0,77 ± 0,95	**0,03**
	FEVE (%)	66,69 ± 5,92	72,05 ± 6,09	**<0,0001**
	Septo (z-score)	+2,18 ± 1,4	+0,61 ± 0,91	**<0,0001**
	Parede posterior do VE (z-score)	+1,74 ± 1,2	+0,4 ± 0,9	**<0,0001**
	Índice de Massa Ventricular Esquerda (IMVE) (g/m^2,7^)	41,74 (17,72 – 108,39)	32,57 (19,07 – 74,7)	**0,0010**
	Frequência de indivíduos com IMVE > P95	24 (43,63%)	0 (0%)	**<0,0001**
**ERP**		**0,44 ± 0,08**	**0,36 ± 0,05**	**<0,0001**
	Frequência de indivíduos com ERP > 0.42	35 (63,63%)	0 (0%)	**<0,0001**
	Diâmetro atrial esquerdo (z-score)	-0,29 ± 0,85	-0,16 ± 1	0,45
	Volume atrial esquerdo (ml/m^2^)	15,63 ± 05,08	16,19 ± 04,54	0,5385
	E Mitral (cm/s)	92,79 ± 21,47	102,91± 17,41	**0,0077**
	A Mitral (cm/s)	61,6 (27,6 – 142)	51,40 (33,8 – 93,6)	**0,0180**
	E/A Mitral (cm/s)	1,57 ± 0,56	1,96 ± 0,51	**0,0003**
	Doppler tecidual e’ septal (cm/s)	10,7 ± 2,61	13,46 ± 2,2	**<0,0001**
	Doppler tecidual e’ lateral e’ (cm/s)	14,2 (6,58 – 29,2)	18,8 (11,5 – 33,1)	**<0,0001**
	E/e’ (cm/s)	6,99 (4,75 – 14,2)	6,38 (3,88 – 11,11)	**0,0092**
**Parâmetros da ecocardiografia com 2DST**				
	*Strain* de reservatório do AE (%)	48,22 ± 10,62	58,52 ± 10,7	**<0,0001**
	*Strain* de condução do AE (%)	37,26 ± 09,77	43,79 ± 10,13	**0,0008**
	*Strain* de contração do AE (%)	11,8 (1,60 – 19,6)	14,30 (5,20 – 27,2)	**0,0009**
	Índice de rigidez atrial esquerda (%^-1^)	0,14 (0,08 – 0,48)	0,11 (0,06 – 0,23)	**<0,0001**
	Índice de enchimento atrial esquerda (cm/s x %^-1^)	2,02 ± 0,63	1,8 ± 0,39	**0,0335**
	*Strain* global longitudinal do pico sistólico do VE (%)	19,4 (9 – 36,4)	21,9 (18,1 – 27,2)	<0,0001

DDVD: diâmetro diastólico do ventrículo direito; DDVE: diâmetro diastólico do ventrículo esquerdo; DSV: diâmetro sistólico do ventrículo esquerdo; VE: ventrículo esquerdo; AE: átrio esquerdo; P95: percentil 95; ERP: espessura relativa de parede; negrito indica p<0,05; variáveis contínuas são expressas como média ± desvio padrão ou mediana (mínimo – máximo) e variáveis categóricas como frequência e porcentagem.

As causas subjacentes de DRC eram anomalias congênitas do rim ou do trato urinário em 34 (61,8%), tubulopatias em sete (12,7%), glomerulopatias em seis (11%) e doenças variadas em oito (14,5%) pacientes. A duração mediana das doenças foi 8,1 (0,83 – 17,5) anos. Havia sete pacientes (12,8%) com DRC estágio I, quatro (7,3%) com DRC estágio II, 12 (21,8%) com DRC estágio III, dois (3,6%) com DRC estágio IV e 30 (54,5%) com DRC estágio V.

Dezenove (34,5%) pacientes não apresentaram hipertensão; 21 (38,2%) apresentaram hipertensão controlada (pressão arterial sistólica e diastólica ≤ percentil 95, em tratamento) e 15 (27,3%) apresentaram hipertensão não controlada (pressão arterial sistólica e diastólica > percentil 95, apesar do tratamento). Os medicamentos anti-hipertensivos incluíram amlodipina (25,5%), enalapril (14,5%), carvedilol (9%), losartana (7,3%), atenolol (3,6%), hidralazina (3,6%) e furosemida (3,6%). Dos pacientes em tratamento para hipertensão, 68% receberam um único agente, 16% receberam dois agentes, e 16% três agentes. Valores medianos de hematócrito foi 35,6% (27,2% - 46,9%), de fósforo sérico foi 4,5mg/dL (2,4mg/dl – 7,2mg/dl) e de PTH 128pg/mL (13 pg/mL - 628 pg/mL). Vite e seis (47,3%) pacientes com DRC apresentaram anemia,^14^ 20 (36,4%) apresentaram concentrações de fósforo acima do limiar esperado^15^ e 33 (60%) apresentaram níveis de PTH acima dos valores alvo.^15^

Entre os 30 pacientes em diálise, 14 (46,7%) faziam hemodiálise e 16 (53,3%) faziam diálise peritoneal. A duração média da diálise foi 2,25 ± 1,2 anos no grupo em hemodiálise e de 1,35 ± 1,09 anos no grupo em diálise peritoneal.

### Ecocardiograma padrão: pacientes com DRC vs. controles

A FEVE foi normal (> 55%) em todos os indivíduos, embora mais baixa nos pacientes que nos controles. O IMVE foi mais alto nos pacientes com DRC. Tanto o diâmetro como o volume do AE foram similares entre os grupos. Apesar de a média da razão E/e’ ter sido maior nos pacientes com DRC, ela foi acima dos limites normais somente em um indivíduo (E/e’ = 14,2)^23^ ( Tabela 1 ). Entre os pacientes com DRC, 14 (25,4%) apresentaram geometria ventricular normal, 17 (30,9%) apresentaram remodelamento concêntrico, 18 (32,7%) apresentaram hipertrofia concêntrica, e seis (11%) hipertrofia excêntrica.

### Ecocardiograma 2DST: pacientes com DRC vs. controles

As imagens obtidas dos pacientes com DRC e dos controles foram satisfatórias; nenhum indivíduo foi excluído da avaliação do *strain* miocárdico. Os pacientes apresentaram valores mais baixos de todos os componentes do *strain* do AE (reservatório, conduto, e contração), rigidez do AE e índice de enchimento mais altos, e *strain* longitudinal global do pico sistólico do VE mais baixos ( Tabela 1 ).

### *Strain* do AE vs. parâmetros ecocardiográficos convencionais nos pacientes com DRC

No grupo de pacientes com DRC, o *strain* de reservatório do AE apresentou correlação negativa com o índice de massa do VE e com E/e’. O *strain* de reservatório do AE correlacionou-se positivamente com o e’ lateral, e’ septal e e’ médio. O índice de enchimento do AE correlacionou-se positivamente com o índice de massa AE, E mitral e E/e’ ( Tabela 2 ).

**Tabela 2:** Correlações entre parâmetros da ecocardiografia convencional e componentes do *strain* atrial esquerdo, índice de rigidez e índice de enchimento do átrio esquerdo nos pacientes com doença renal crônica

	*Strain* longitudinal de reservatório do átrio esquerdo (%)	*Strain* longitudinal de condução do átrio esquerdo (%)	*Strain* longitudinal de contração do átrio esquerdo (%)	Índice de rigidez do átrio esquerdo	Índice de enchimento do átrio esquerdo
FEVE (%)	0,12 (0,3748)	0,12 (0,4024)	0,01 (0,9272)	-0,10 (0,4496)	0,01 (0,9241)
IMVE (g/m^2,7^)	-0,48 (0,0002)	**-0,42 (0,0016)**	-0,18 (0,1994)	0,50 (0,0001)	**0,37 (0,0059)**
ERP	-0,13 (0,3263)	-0,10 (0,4680)	-0,15 (0,2861)	0,11 (0,4391)	0,13 (0,3388)
Volume do AE (mm/m^2^)	-0,03 (0,8013)	-0,11 (0,4357)	0,10 (0,4686)	-0,04 (0,7459)	0,10 (0,4507)
E Mitral (cm/s)	-0,10 (0,4659)	0,11 (0,4355)	**-0,28 (0,0370)**	**0,35 (0,0082)**	**0,70 (<0,0001)**
A Mitral (cm/s)	-0,14 (0,3204)	-0,12 (0,3945)	0,00 (0,9860)	**0,29 (0,0347)**	0,25 (0,0668)
E/A Mitral (cm/s)	0,11 (0,4307)	0,21 (0,1225)	-0,15 (0,2765)	-0,11 (0,4359)	0,16 (0,2354)
Doppler tecidual e’ lateral (cm/s)	**0,46 (0,0004)**	**0,46 (0,0004)**	0,10 (0,4573)	**-0,57 (<0,0001)**	-0,21 (0,1328)
Doppler tecidual e’ septal (cm/s)	**0,34 (0,0106)**	**0,40 (0,0027)**	-0,02 (0,8863)	**-0,32 (0,0185)**	0,13 (0,3585)
e’ médio (cm/s)	**0,49 (0,0001)**	**0,50 (0,0001)**	0,08 (0,5658)	**-0,56 (<0,0001)**	-0,09 (0,4922)
E/e’ (cm/s)	-0,48 (0,0002)	-0,30 (0,0251)	-0,33 (0,0142)	0,83 (<0,0001)	**0,73 (<0,0001)**

FEVE: fração de ejeção do ventrículo esquerdo; IMVE: índice de massa ventricular esquerda; ERP: espessura relativa da parede; AE: átrio esquerdo; os dados são apresentados como coeficiente de correlação de Spearman (valor p); negrito indica p<0,05.

### *Strain* do AE vs. *strain longitudinal* global do pico sistólico do VE nos pacientes com DRC

**Figura 2:**
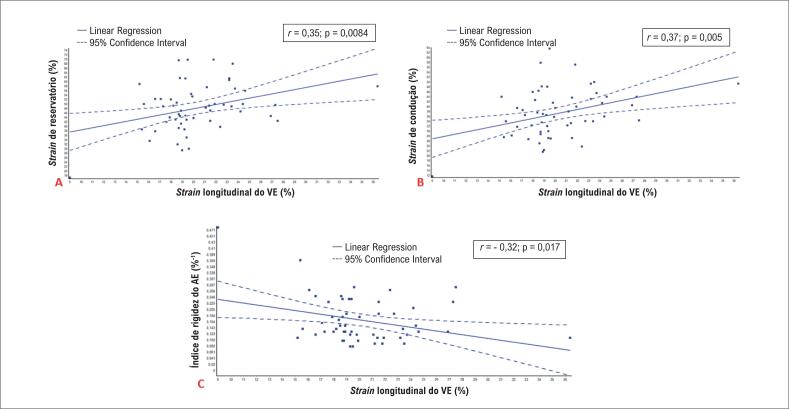
Correlações entre o *strain* longitudinal global do pico sistólico do ventrículo esquerdo e parâmetros do *strain* do átrio esquerdo; VE: ventrículo esquerdo.

O *strain* longitudinal global do pico sistólico do VE correlacionou-se positivamente com o *strain* de reservatório e com o *strain* de condução, e negativamente com o índice de rigidez do AE ( Figura 2 ).

### Parâmetros do ecocardiograma com 2DST de acordo com o estágio da DRC

**Figura 3:**
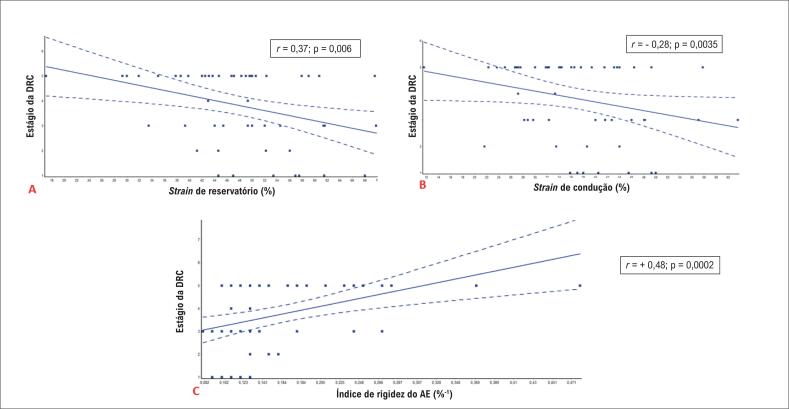
Parâmetros de strain do átrio esquerdo de acordo com o estágio da doença renal crônica (DRC).

Observou-se uma correlação negativa do estágio da DRC com o *strain* de reservatório do AE e do *strain* de condução. Além disso, houve uma correlação positiva moderada entre o estágio da DRC e o índice de rigidez do AE ( Figura 3 ). Não houve correlação do estágio da DRC com o *strain* de contração do AE, o índice de enchimento do AE ou o *strain* longitudinal global do pico sistólico do VE.

### Parâmetros do ecocardiograma com 2DST de acordo com o índice de massa do VE na DRC

Os pacientes com DRC e índice de massa do VE acima do percentil 95 apresentaram valores mais baixos de *strain* de reservatório e de *strain* de condução, e valores mais altos do índice de rigidez e de índice de enchimento. O *strain* de contração do AE e o *strain* longitudinal global do pico sistólico do VE foram similares entre os grupos ( Tabela 3 ).

**Tabela 3:** Ecocardiografia bidimensional com *speckle tracking* (2DST): pacientes com doença renal crônica e hipertrofia ventricular esquerda vs. pacientes com doença renal crônica sem hipertrofia ventricular esquerda

Ecocardiograma com 2DST	IMVE ≤ P95 (n=31)	IMVE > P95 (n=24)	Valor p
*Strain* de reservatório do AE (%)	52,99 ± 9,52	42,05 ± 8,74	<0,0001
*Strain* de condução do AE (%)	41 ± 9,63	32,43 ± 7,74	0,0005
*Strain* de contração do AE (%)	12,5 (4,2 – 19,6)	9,3 (1,6 – 16,2)	0,1144
Índice de rigidez atrial esquerda (%^-1^)	0,13 (0,08 – 0,23)	0,23 (0,11 – 0,48)	<0,0001
Índice de enchimento atrial esquerdo (cm/s x %^-1^)	1,74 ± 0,47	2,39 ± 0,63	0,0001
*Strain* global longitudinal do pico sistólico do VE (%)	19,4 (15,2 – 36,4)	19,35 (9 – 27,5)	0,4152

P95: percentil 95; negrito indica p<0,05; dados contínuos são apresentados como média ± desvio padrão ou mediana (mínimo-máximo); IMVE: índice de massa ventricular esquerda; VE: ventrículo esquerdo.

### Parâmetros do ecocardiograma com 2DST de acordo com a geometria na DRC

O *strain* longitudinal global do pico sistólico do VE foi similar nos quatro grupos de geometria do VE. Comparando os pacientes com hipertrofia concêntrica e pacientes com geometria normal do VE, o primeiro grupo apresentou valores de *strain* de reservatório (40,97 ± 9,53% *vs* . 54,36 ± 8,6%) e *strain* de condução (32,23 ± 8,59% vs. 41,89 ± 9,32%)mais baixos, e valores mais altos de índice de rigidez do AE [0,23 (0,11–0,48) %^-1^ *vs* . 0,12 (0,08–0,23) %^-1^] e índice de enchimento do AE (2,43 ± 0,59 cm/s x %^-1^ vs. 1,73 ± 0,49 cm/s x %^-1^) (p<0,05).

Além disso, comparando os pacientes com hipertrofia concêntrica e pacientes com remodelamento concêntrico, o primeiro grupo apresentou *strain* de reservatório mais baixo (40,97 ± 9,53% *vs* . 51,86 ± 10,34%), e valores mais altos de índice de rigidez do AE [0,23 (0,11 – 0,48) %^-1^ *vs,* 0,13 (0,08 – 0,19) %^-1^] e de índice de enchimento do AE (2,43 ± 0,59 cm/s x %^-1^ vs, 1,74±0,46 cm/s x %^-1^) (p<0,05).

Não houve diferenças significativas entre os parâmetros do *strain* do AE comparando-se pacientes com hipertrofia concêntrica e pacientes com hipertrofia excêntrica do VE.

### Parâmetros do ecocardiograma com 2DST de acordo com o controle da pressão arterial na DRC

Pacientes com DRC e hipertensão não controlada apresentaram *strain* de reservatório e *strain* de condução do AE mais baixos. O *strain* longitudinal global do pico sistólico do VE também foi reduzido no grupo de pacientes com hipertensão não controlada ( Tabela 4 ).

**Tabela 4:** Ecocardiografia bidimensional com *speckle tracking* (2DST): pacientes com doença renal crônica com pressão normal ou hipertensão controlada vs. pacientes com doença renal crônica e hipertensão não controlada

Ecocardiograma com 2DST	Sem hipertensão / hipertensão controlada (n=40)	Hipertensão n ão controlada (n=15)	Valor p
*Strain* de reservatório do AE (%)	50,6 ± 9,7	41,9 ± 10,6	0,0055
*Strain* de condução do AE (%)	38,30 (22,3 – 63,60)	32,00 (11,60 – 48,20)	0,0190
*Strain* de contração do AE (%)	12,10 (1,60 – 19,40)	10,00 (3,00 – 19,60)	0,3304
Índice de rigidez do átrio esquerdo (%^-1^)	0,14 (0,08 – 0,28)	0,15 (0,10 – 0,48)	0,1695
Índice de enchimento atrial esquerdo (cm/s x %^-1^)	1,93 ± 0,57	2,27 ± 0,72	0,1093
*Strain* global longitudinal do pico sistólico do VE (%)	19,85 (15,20 – 36,40)	18,80 (9,00 – 24,20)	0,0482

P95: percentil 95; negrito indica p<0,05; dados contínuos são apresentados como média ± desvio padrão ou mediana (mínimo-máximo).

### Comparações entre pacientes com DRC dialíticos e pacientes com DRC não dialíticos

Pacientes com DRC dialíticos e não dialíticos tinham idade e distribuição de gêneros similares. Pacientes dialíticos apresentaram peso seco, altura e ASC mais baixos.

Parâmetros da ecocardiografia convencional e com 2DST estão apresentados na Tabela 5 . O IMVE e a razão E/e’ eram maiores nos pacientes dialíticos e a FEVE e o volume do AE foram similares entre os grupos. Observou-se uma associação significativa entre a geometria do AE anormal e diálise. Somente um dos cinco pacientes com hipertrofia excêntrica não estava em diálise. Apesar disso, a associação entre hipertrofia excêntrica e diálise não foi significativa, provavelmente devido ao número pequeno de pacientes na amostra (p=0,20). O *strain* de reservatório do AE foi mais baixo, e o índice de rigidez do AE foi mais alto no grupo de pacientes dialíticos.

**Tabela 5:** Pacientes com doença renal crônica não dialíticos vs. dialíticos: dados demográficos, parâmetros da ecocardiografia convencional e parâmetros da ecocardiografia bidimensional com *speckle tracking* (2DST)

Dados demográficos		Não dialítico (n=25)	Dialítico (n=30)	Valor-p
Idade (anos)		10,6 ± 4,1	8,5 ± 5	0,1100
Sex (male)		13 (52,00%)	23 (76,67%)	0,1029
Peso seco (kg)		31,00 (14,50 – 100,00)	18,05 (05,00 – 73,00)	0,0075
Altura (m)		01,04 (01,00 – 01,85)	01,11 (00,66 – 01,67)	**0,0026**
ASC (m^2^)		01,12 ± 00,39	00,85 ± 00,41	**0,0046**
Frequência cardíaca (bpm)		100 ± 13	105 ± 14	**0,1800**
**Parâmetros da ecocardiografia convencional**				
	DDVD (z-score)	-0,78 ± 1,13	-0,11 ± 1,35	0,0540
	DDVE (z-score)	-0,62 ± 1,14	-0,05 ± 1,37	0,1070
	FEVE (%)	66,35 ± 6,48	66,97 ± 5,51	0,7082
	Septo (z-score)	+1,74 ± 1,21	+2,5 ± 1,48	**0,0363**
	Parede posterior do VE (z-score)	+1,30 ± 0,97	+2,1 ± 1,27	**0,0127**
	Índice de Massa Ventricular Esquerda (IMVE) (g/m^2,7^)	32,37 (17,72 – 54,1)	51,6 (21,58 – 108,39)	**<0,0001**
	Frequência de indivíduos com IMVE > P95	6 (24,00%)	18 (60,00%)	**0,0160**
	ERP	0,43 (0,31 – 0,64)	0,46 (0,25 – 0,63)	0,2452
	Frequência de indivíduos com ERP > 0.42	14 (56,00%)	21 (70,00%)	0,4276
	Frequência de geometria do VE anormal	15 (60%)	26 (86,6%)	**0,032**
	Diâmetro atrial esquerdo (z-score)	-0,19 ± 0,91	-0,38 ± 0,81	0,3974
	Volume atrial esquerdo (ml/m^2^)	16,18 (9,15 – 28,57)	13 (9,13 – 24)	0,1369
	E Mitral (cm/s)	92,98 ±16,51	92,64 ± 25,16	0,9524
	A Mitral (cm/s)	55,1 (27,6 – 102)	64,45 (36,9 – 142)	**0,0006**
	E/A Mitral (cm/s)	1,87 ± 0,59	1,33 ± 0,41	**0,0003**
	Tissue Doppler septal e’ (cm/s)	11,99 ± 2,25	9,63 ± 2,42	**0,0004**
	Tissue Doppler lateral e’ (cm/s)	16,66 ± 3,61	13,11 ± 3,58	**0,0006**
	E/e’ (cm/s)	6,3 (4,75 – 9,54)	7,89 (5,47 – 14,2)	**0,0007**
**Parâmetros da ecocardiografia com 2DST**				
	*Strain* de reservatório do AE (%)	52,24 ± 9,58	44,87 ± 10,42	**0,0086**
	*Strain* de condução do AE (%)	40,0 2 ± 9,82	34,96 ± 9,26	0,0563
	*Strain* de contração do AE (%)	12,2 (4,2 – 19,6)	9,8 (1,6 – 17,5)	0,1281
	Índice de rigidez atrial esquerda (%^-1^)	0,13 ± 0,05	0,20 ± 0,08	**0,0005**
	Índice de enchimento atrial esquerdo (cm/s x %^-1^)	1,86 ± 0,55	2,15 ± 0,66	0,0766
	*Strain* global longitudinal do pico sistólico do VE (%)	19,4 (16,1 – 26,9)	19,35 (9 – 36,4)	0,7738

DDVD: diâmetro diastólico do ventrículo direito; DDVE: diâmetro diastólico do ventrículo esquerdo; DSV: diâmetro sistólico do ventrículo esquerdo; VE: ventrículo esquerdo; AE: átrio esquerdo; P95: percentil 95; ERP: espessura relativa de parede; negrito indica p<0,05; variáveis contínuas são expressas como média ± desvio padrão ou mediana (mínimo – máximo) e variáveis categóricas como frequência e porcentagem.

### Comparações entre pacientes em diálise peritoneal e hemodiálise

Os parâmetros de *strain* atrial esquerdo não foram diferentes entre pacientes em diálise peritoneal e pacientes em hemodiálise ( Tabela 6 ).

**Tabela 6:** Peritoneal vs. hemodiálise: parâmetros do *speckle-tracking*

Parâmetros de strain	Peritoneal (n=14)	Hemodiálise (n=16)	Valor p
*Strain* global longitudinal do pico sistólico do VE (%)	21,20±6,47	19,55±2,83	0,3894
*Strain* de reservatório do AE (%)	41,36±10,41	47,94±9,72	0,0859
*Strain* de condução do AE (%)	31,63±9,24	37,88±8,52	0,0661
*Strain* de contração do AE (%)	8,40 (4,50 – 16,00)	12,15 (1,60 – 17,50)	0,9834
Índice de rigidez atrial esquerda (%^-1^)	0,21 (0,11 – 0,48)	0,18 (0,10 – 0,28)	0,3816
Índice de enchimento atrial esquerdo (cm/s x %^-1^)	2,29 (1,25 – 2,95)	2,14 (0,88 – 3,33)	0,6100

AE: átrio esquerdo; VE: ventrículo esquerdo.

### Variabilidade intra-observador e entre observadores

Obteve-se CCI adequado (>0,80) para todos os parâmetros ecocardiográficos com 2DST para variabilidade intra-observador e entre observadores, exceto para *strain* contrátil do AE (CCI=0,61 para variabilidade) ( Tabela 7 ). Os principais resultados do estudo são destacados na Figura Central .

**Tabela 7:** Variabilidade intra-observador e entre observadores dos parâmetros do *speckle-tracking*

Parâmetros	Teste intra-observador		Teste entre obseradores	
	CCI (IC)	Valor p	CCI (IC)	Valor p
*Strain* de reservatório do AE (%)	0,99 (0,98 – 1,00)	<0,0001	0,83 (0,57 – 0,93)	<0,0001
*Strain* de condução do AE (%)	0,99 (0,98 – 1,00)	<0,0001	0,87 (0,67 – 0,95)	<0,0001
*Strain* de contração do AE (%)	0,92 (0,81 – 0,97)	<0,0001	0,61 (0,03 – 0,84)	<0,0001
*Strain* global longitudinal do pico sistólico do VE (%)	0,98 (0,94 – 0,99)	<0,0001	0,89 (0,74 – 0,96)	<0,0001

CCI: coeficiente de correlação intraclasse; AE: átrio esquerdo; VE: ventrículo esquerdo.

## Discussão

Este estudo se destaca pela detecção de disfunção subclínica da deformação atrial esquerda em crianças com DRC e diferentes estágios da doença, com grande viabilidade e reprodutibilidade. Além disso, foi possível demonstrar associações significativas entre a disfunção na deformação do AE e fatores de risco previamente demonstrados em pacientes com DRC, tais como hipertrofia do VE e hipertensão sistêmica não controlada.

Estudos anteriores usando imagens por Doppler tecidual sugeriram parâmetros de disfunção diastólica do VE no início da progressão da DRC, sendo os piores valores registrados nos pacientes em diálise de manutenção.^24^ No entanto, somente um paciente com DRC em nosso estudo mostrou um E/e’ médio superior a 14, um dos principais marcadores não invasivos de disfunção diastólica em pacientes com fração de ejeção preservada, de acordo com as diretrizes da ASE para adultos.^23^

Há evidência crescente de que algoritmos atuais para a avaliação de disfunção diastólica em adultos não sejam tão confiáveis em populações pediátricas. Além disso, em crianças com vários tipos de cardiomiopatias, os critérios para disfunção diastólica foram discrepantes na maioria dos pacientes, e metade deles apresentaram valores de E/e’ dentro da faixa de normalidade para idade.^5^ Similar ao estudo de Morris et al.,^25^ nossos dados corroboram que o *strain* de deformação do AE e o índice de rigidez do AE sejam parâmetros diastólicos adicionais, com valor prognóstico que necessita ainda de comprovação em pacientes pediátricos.^25^

Apesar da redução significativa no *strain* de reservatório, o volume atrial esquerdo na nossa população pediátrica com DRC e o grupo controle eram similares. De fato, recentemente foi demonstrado que mudanças no *strain* de reservatório do AE precedem o aumento no volume atrial esquerdo, classicamente conhecido como um marco de disfunção diastólica.^26^ Estudos com pacientes adultos com DRC mostraram uma correlação inversa entre o *strain* atrial esquerdo e a pressão capilar pulmonar média, independentemente do volume do AE.^27^ Nakanishi et al.^28^ sugeriram diferentes mecanismos que pudessem estar envolvidos no desenvolvimento da disfunção atrial esquerda na DRC e volume atrial esquerdo ainda normal: estado inflamatório crônico, fibrose miocárdica induzida por ativação crônica do sistema renina-angiotensina-aldosterona, estímulo simpático e estresse oxidativo.^28^

Dados publicados sobre a função diastólica em pacientes pediátricos com DRC em diferentes estágios da doença são escassos. Em nosso estudo, a DRC correlacionou-se negativamente com o *strain* de reservatório, e positivamente com o índice de rigidez do AE, sugerindo que esses novos parâmetros possam refletir a progressão da doença renal e a piora da função diastólica, mesmo na ausência de uma insuficiência cardíaca clara. De fato, Gan et al.^29^ demonstraram o valor prognóstico do strain de reservatório do AE como um preditor independente da progressão da disfunção renal em adultos com DRC estágio 3 ou 4, sem história de doença cardíaca prévia e com função renal estável.^29^

Apesar de nossos pacientes com e sem IMVE acima do percentil 95 tenham apresentado valores similares de *strain* sistólico do VE, a disfunção do *strain* atrial esquerdo foi significativamente associada com hipertrofia do VE. Ainda, os pacientes com DRC e hipertrofia concêntrica apresentaram *strain* de reservatório mais baixo, e índice de rigidez e índice de enchimento do AE mais altos em comparação aos pacientes com DRC e geometria normal do VE ou remodelamento concêntrico. Essa informação parece clinicamente relevante, uma vez que a hipertrofia do VE é o indicador mais importante de risco cardiovascular na população com DRC e padrões anormais da geometria do VE afeta adversamente o prognóstico.^30 – 32^

A hipertensão não controlada em nossos pacientes com DRC foi frequente (27,3%) e associada com *strain* de reservatório atrial esquerdo mais baixo e índice de rigidez mais alto. Esses achados podem ter um impacto sobre o prognóstico, já que um estudo recente de Zhao et al.^33^ demonstrou que o índice de rigidez do AE precede a hipertrofia do VE, além de ser independentemente correlacionado com lesão em órgão alvo em pacientes adultos com hipertensão.

Tradicionalmente, as funções sistólica e diastólica são avaliadas como fases separadas. Contudo, elas são intimamente relacionadas por vários mecanismos, como o mecanismo de Frank-Starling, em que um maior enchimento aumenta a contratilidade, o que, por sua vez, aumenta a retração elástica no início da diástole. Corroborando estudos anteriores que descreveram o acoplamento sistólico e diastólico, nós documentamos, em nosso grupo de crianças com DRC, uma correlação significativa do *strain* longitudinal global do pico sistólico com o *strain* de reservatório, o *strain* de condução e o índice de rigidez do AE.^34^

Em comparação aos pacientes não dialíticos, nossos pacientes dialíticos apresentaram um *strain* de reservatório mais baixo e um índice de rigidez atrial esquerdo mais alto. O impacto da diálise sobre a função diastólica também foi investigado por Doan et al.^35^ que avaliaram o *strain* atrial esquerdo antes, durante e após sessões de hemodiálise. Os autores descreveram uma redução significativa do strain atrial esquerdo no meio da diálise, com retorno aos valores basais após a diálise.^35^

### Limitações do estudo

Possíveis limitações do estudo são o pequeno número de pacientes incluídos e o caráter unicêntrico do estudo, que podem impedir a generalização das conclusões para populações maiores. Uma vez que somos um centro de referência em nefrologia pediátrica, a alta prevalência da doença renal terminal em nossa amostra (54% no estágio V) pode haver contribuído para a identificação de deformações mais acentuadas do AE.

Ecocardiogramas convencional e com 2DST foram analisados pelo mesmo cardiologista pediátrico, cego para os prontuários médicos. Esse examinador, porém, tinha conhecimento sobre quais participantes eram pacientes e quais eram controle. Contudo, o segundo observador era absolutamente cego para a alocação do grupo e o CCI foi considerado adequado.

Os pacientes em diálise foram examinados mais próximos de seus pesos secos clinicamente estimados, uma vez que não avaliamos a volemia.

Não incluímos níveis séricos de pró-peptídeo natriurético cerebral (pro-BNP) ou de mediadores da inflamação no presente estudo, uma vez que eles não são solicitados de maneira rotineira pelos médicos de nossos ambulatórios. Além disso, nós não investigamos possíveis correlações entre o *strain* atrial esquerdo e a capacidade de exercício de nossos pacientes pediátricos com DRC. Tudo isso teria auxiliado a detectar disfunções sutis no miocárdio associadas com o comprometimento do *strain* atrial esquerdo.

Embora a hemodiálise esteja geralmente associada com maior comprometimento cardiovascular que a diálise peritoneal,^33^ não encontramos diferença significativa dos parâmetros de strain do AE entre esses dois tipos de terapia renal substitutiva, talvez devido ao pequeno tamanho amostral em cada grupo de pacientes.

Como nosso estudo foi delineado como um estudo transversal, não foram investigadas as implicações prognósticas da avaliação do *strain* atrial esquerdo nos pacientes pediátricos com DRC, incluindo morbidade e mortalidade.

## Conclusão

A avaliação do *strain* do AE mostrou-se uma ferramenta viável para a avaliação diastólica em uma população de pacientes pediátricos com DRC. O presente estudo documentou associações significativas entre o comprometimento do *strain* do AE e fatores de risco cardiovasculares nessa população. Como a disfunção diastólica tem um forte valor prognóstico na DRC, a incorporação do *strain* atrial esquerdo na avaliação ecocardiográfica nesta população parece ser uma estratégia apropriada.

A avaliação longitudinal usando nesses novos índices não invasivos podem revelar os efeitos da DRC sobre a saúde cardiovascular em longo prazo ao longo do desenvolvimento da criança.
