# Supplementary material for: Strain do Átrio Esquerdo pela Técnica de Speckle-Tracking : Contribuição para a Avaliação da Função Diastólica em Pacientes Pediátricos com Doença Renal Crônica
Source: Arq Bras Cardiol. 2024 Apr 12;121(3):e20230131. [Article in Portuguese] doi: 10.36660/abc.20230131 (PMC12092048; doi:10.36660/abc.20230131)
Supplement: Translation

# Translation: Speckle-Tracking: Incremental Role in Diastolic Assessment of Pediatric Patients with Chronic Kidney Disease

**DOI:** 10.36660/abc.20230131i

**Authors:** Flora Maciel Penachio, Maria de Fátima Rodrigues Diniz, Rosana Sbruzzi Prado Laurino, Andreia Watanabe, Karen Saori Shiraishi Sawamura, Alessandro Cavalcanti Lianza, Carolina Rocha Brito Menezes, Isabela de Sousa Lobo Silva, Gabriela Nunes Leal

**Affiliations:** 1 São Paulo SP Brazil Universidade de São Paulo – Faculdade de Medicina, São Paulo, SP – Brazil; 2 São Paulo SP Brazil Instituto da Criança da Universidade de São Paulo, São Paulo, SP – Brazil; 3 São Paulo SP Brazil Hospital das Clínicas da Universidade de São Paulo, São Paulo, SP – Brazil; 4 São Paulo SP Brazil Hospital do Coração, São Paulo, SP – Brazil; 5 São Paulo SP Brazil Hospital Israelita Albert Einstein, São Paulo, SP – Brazil; 6 São Paulo SP Brazil Hospital Sírio Libanês, São Paulo, SP – Brazil

**Keywords:** Heart Atria, Kidney, Echocardiography, Child

## Abstract

**Background:**

Cardiovascular complications are the leading cause of mortality in pediatric patients with chronic kidney disease (CKD). Echocardiographic assessment of diastolic function in CKD has been limited to spectral and tissue Doppler imaging, known to be less reliable techniques in pediatrics. Two-dimensional Speckle tracking echocardiography (2DST) derived left atrial (LA) strain has recently been confirmed as a robust measure of diastolic function.

**Objectives:**

To investigate LA strain role in diastolic assessment of children at different stages of CKD.

**Methods:**

From February 2019 to July 2022, 55 CKD patients without cardiovascular symptoms and 55 controls were evaluated by standard and 2DST echocardiograms. The level of significance was set at 5% (p<0.05).

**Results:**

Patients and controls had similar age [9.78 (0.89 – 17.54) vs. 10.72 (1.03 –18,44) years; p = 0.41] and gender (36M:19F vs. 34M:21F; p=0.84). There were 25 non-dialysis patients and 30 dialysis patients. Left ventricular ejection fraction was ≥ 55% in all of them. Comparing CKD and controls, LA reservoir strain was lower (48.22±10.62% vs. 58.52±10.70%) and LA stiffness index was higher [0.14 (0.08–0.48)%^-1^vs. 0.11 (0.06–0.23) %^-1^]; p<0.0001. LV hypertrophy was associated with lower LA reservoir strain (42.05±8.74% vs. 52.99±9.52%), higher LA stiffness [0.23(0.11 – 0.48)%^-1^vs. 0.13 (0.08–0.23) %^-1^and filling indexes (2.39±0.63 cm/s x %^-1^vs. 1.74±0.47 cm/s x %^-1^; p<0.0001. Uncontrolled hypertension was associated with lower LA reservoir strain (41.9±10.6% vs. 50.6±9.7; p=0.005).

**Conclusions:**

LA strain proved to be a feasible tool in the assessment of pediatric CKD patients and was associated with known cardiovascular risk factors.

## Introduction

Cardiovascular complications are the leading cause of mortality among children and adolescents with chronic kidney disease (CKD), being responsible for up to 30% of deaths in this population.^1^These data are in sharp contrast to the general pediatric population, in which cardiovascular disease mortality is very low, accounting for less than 3% of all deaths. Despite advances in renal substitutive therapy, mortality rates due to cardiovascular diseases in CKD pediatric patients had not changed significantly over the last decades.^2^

Myocardium remodeling in CKD has traditionally been understood as a physiologic adaptation to reduce ventricular wall stress in response to volume overload and hypertension. However, there are several additional factors that contribute to left ventricular (LV) remodeling and diastolic dysfunction, such as uremic toxins, anemia, FGF23, high serum levels of phosphorus, hyperparathyroidism and fibrosis induced by oxidative stress, and by activation of the renin-angiotensin-aldosterone system.^3^

LV diastolic dysfunction is common in CKD patients and has been linked to poor cardiovascular outcomes.^4^Nevertheless, most of the published literature on the assessment of diastolic function in CKD children has been limited to spectral and tissue Doppler imaging, known to be less reliable techniques in pediatrics. A recent study by Dragulescu et al.^5^demonstrated that diastolic parameters derived from adult studies are inadequate and not sufficiently discriminatory in childhood.^5^Moreover, the large range of normal pediatric reference values allows the diagnosis of diastolic disfunction in only a small proportion of patients.^6^

Given its dynamic relationship with LV function, the left atrium reflects changes in LV filling pressures, making it a sensitive surrogate marker of diastolic dysfunction.^7^Two-dimensional Speckle tracking echocardiography (2DST) evaluation of left atrial (LA) strain has recently been confirmed as a robust measure of LA function, in different clinical scenarios.^8^The left atrium plays a critical role in maintaining LV filling by functioning as a reservoir for pulmonary venous flow during LV systole, a conduit for blood flow into the LV during early diastole and as a booster pump during late diastole.^8^Alterations in LA reservoir strain precedes changes in LA volume, favoring its use to detect subclinical diastolic dysfunction.^9^LA stiffness index, calculated as ratio of E/e' to LA reservoir strain, was able to differentiate children with cardiomyopathy from healthy controls with good accuracy.^10^LA filling index, calculated as ratio of mitral E to LA reservoir strain, showed better diagnostic performance to determine elevated LV filling pressure than E/e’. Furthermore, a recent work demonstrated that LA reservoir strain was an independent predictor of cardiovascular death and adverse events in adult CKD patients.^11 , 12^

Since the assessment of diastolic function by echocardiogram remains a challenge in pediatrics, with lack of gold standard parameters, incorporation of new modalities such as LA considerations, the present study aimed to investigate the role of LA strain in the assessment of diastolic function in children and adolescents at different stages of CKD.

## Methods

### Study design and population

From February 2019 to July 2022, 55 CKD consecutive outpatients were recruited during their routine visits to our Pediatric Nephrology Unit. None of them showed symptoms of heart failure (New York Heart Association class I) and congenital heart diseases had been ruled out by previous echocardiographic evaluations. Exclusion criteria included inadequate quality of image or refusal to participate in the study. The control group comprised 55 healthy volunteers from primary care clinics, with no history of cardiovascular disease and with normal echocardiograms. The ethics committee of our institution approved this cross-sectional study, and written informed consent was obtained from all participants and their legal guardians.

Patients’ medical records were carefully reviewed for demographic and clinical data by the attendant physician, by the time of the echocardiogram. Demographic data included age, gender, dry weight, height, and body surface area (BSA), calculated by the Haycock formula.^13^Clinical data included CKD etiology, presence, type and duration of dialysis, presence of hypertension, cardiovascular medications in use, hematocrit,^14^and phosphorus^15^and parathyroid hormone levels.^15^According to recommendations of the task force, hypertension was defined when systolic and/or diastolic blood pressure was >95^th^percentile for the child's age, sex, and height.^16^CKD classification was based on glomerular filtration rate (GFR), estimated by Schwartz formula: stage I (GRF > 90 ml/min/1.73 m^2^); stage II (GFR between 60 and 89 ml/min/1.73 m^2^); stage III (GFR between 30 and 59 ml/min/1.73 m^2^); stage IV (GFR between 15 and 29 ml/min/1.73 m^2^) and stage V (GFR < 15 ml/min/1.73 m^2^).^17^

Standard and 2DST echocardiograms were obtained by the same pediatric cardiologist, blinded to medical records. The examiner was, however, aware of the subjects as either patients or controls. Dialysis patients were evaluated from four to six hours after the last session.

### Standard echocardiogram

Standard transthoracic echocardiography was performed according to the recommendations of the American Society of Echocardiography (ASE) and included M-mode, two-dimensional imaging, conventional, and tissue Doppler evaluation at the septal and lateral mitral annulus.^18^The equipment used was a Philips Affiniti 70 (Andover, MA 01810 USA), with multifrequency transducers (S 5-1 and S 8-3 MHz). Cardiac chamber dimensions were obtained in two-dimensional mode, and left ventricle ejection fraction (LVEF) was calculated by Simpson's method. Cardiac chambers’ diameters, as well as septum and posterior wall thickness, were expressed as z-score values.^19^LV mass (g) was estimated using the Devereaux's formula according to the Penn convention and indexed for height (m) raised to an exponential power of 2.7.^18^LV mass index (LVMI) percentile was calculated for each patient, according to age-specific reference intervals proposed by Khoury et al.^20^LV relative wall thickness (RWT) was calculated as the sum of septum and posterior wall thickness divided by LV diastolic diameter (normal value ≤ 0.42). LV geometry was then classified as concentric remodeling (abnormal RWT and normal LVMI), concentric hypertrophy (abnormal RWT and LVMI) and eccentric LV hypertrophy (abnormal LVMI and normal RWT).^20^

Evaluation of LV diastolic function included both conventional and tissue Doppler-based measurements – mitral E and A velocities, E/A ratio, and E/e’ ratio, with e’ being the average of values obtained by tissue Doppler at the septal and lateral annulus. Left atrial volume was estimated using the biplane area-length method, at end-ventricular systole, and values were indexed to the BSA.^18^

### 2DST echocardiogram

LA-focused two-dimensional cine-loop recordings were obtained from apical four chamber view and digitally stored for offline speckle-tracking strain analysis by a dedicated software (Q Lab 15, Philips Medical Systems). The frame rate was set between 80 and 90 frames/s to ensure adequate speckle-tracking. Care was taken to obtain true apical images, avoiding foreshortening. In segments with insufficient tracking, manual readjustment of the endocardial border was applied to optimize tracking quality. The LA tracing for strain was terminated 0.5 cm above the atrioventricular junction, to avoid influence of mitral annular motion.^21^The onset of R-wave on the electrocardiogram was used as zero-reference point of the strain analysis. LA reservoir strain was defined as the peak systolic strain, just before mitral valve opening. This was followed by a plateau and a second late peak at the onset of the P-wave indicating the contractile strain. Conduit strain was calculated as the difference between reservoir and contractile strain^21^( Figure 1 ). LA stiffness index was calculated as ratio of E/e' to LA reservoir strain^10^and LA filling index as ratio of mitral E to LA reservoir strain.^11^

**Figure 1:** Left atrium strain components. A: control. B: Chronic Kidney Disease (CKD) patient. LASr: reservoir strain; LAScd: conduit strain. All components are reduced in CKD; LV: left ventricle; LA: left atrium.

To evaluate global longitudinal LV systolic strain, two-dimensional cine-loop recordings of apical, four-, three-, and two-chamber views were acquired and digitally stored for analysis. A sector scan angle of 30 - 60° and frame rates of 80–90 Hz were chosen. The endocardial tracing was automatically generated by the computer algorithm (Q Lab 15, Philips Medical Systems) and manually adjusted when necessary. Global LV peak systolic global longitudinal strain was calculated, representing the average values of the 17 ventricular segments analyzed in the three views.^22^

### Statistical analysis

Statistical analyses were performed using R software with the R Studio integrated development environment (Version 4.1.0, RStudio, Inc).

Categorical data were presented as absolute and relative frequencies and continuous data as mean ± standard deviation (sd) or median (range). The Kolmogorov-Smirnov test was used to verify the normality of data. Unpaired Student's t test was used to assess normally distributed continuous data and Mann-Whitney test to assess non-normally distributed continuous data. One-way ANOVA was used to compare more than two groups for variables with normal distribution; Kruskal-Wallis was chosen for non-normally distributed variables. In both situations, multiple comparisons were conducted in the post hoc test applying Bonferroni procedure.

Chi-square test was used to compare categorical data. Spearman's correlation coefficient was used to investigate the relationships between 2DST and standard echocardiographic parameters. The level of significance was set at 5% (p < 0.05).

Intra- and interobserver variability was tested, regarding 2DST measurements. The first examiner repeated the analysis of 20 CKD patients and 20 healthy controls randomly selected, three months after image acquisition. Randomization of participants consisted of drawing a number (their registration numbers) from a box.

A second observer, unaware of previous results, also performed offline analysis of the same individuals.

Intra- and interobserver variability for strain measurements was assessed using intraclass correlation coefficient (ICC), with good correlation being defined as ICC > 0.8.

## Results

### Demographic and clinical data

CKD patients and controls had similar age (9.78 [0.89 – 17.54] years vs. 10.72 [1.03 – 18.44] years; p=0.41) and gender distribution (36M:19F vs. 34M:21F; p=0.84). As expected, dry weight, height and BSA were significantly lower among CKD patients ( Table 1 ).

**Table 1:** Chronic kidney disease patients vs. controls: demographic data, standard and two-dimensional speckle tracking (2DST) echocardiography parameters

Demographic data		CKD (n=55)	Control (n=55)	p-value
Age (years)		9.5 ± 4.9	10.4 ± 5	0.3878
Gender (male)		36 (65.45%)	34 (61.81%)	0.8429
Dry Weight (kg)		25 (5 – 100)	43 (10 – 84)	0.0009
Height (m)		1.29 (0.66 – 1.85)	1.45 (0.74 – 1.84)	0.0053
BSA (m^2^)		0.97 ± 0.42	1.25 ± 0.44	**0.0009**
Heart rate (bpm)		85 ± 14	90 ± 25	**0.1900**
**Standard echocardiographic parameters**				
	LVDD (z-score)	-0.41 ± 1.3	-0.22 ± 1.0	0.39
	LVSD (z-score)	-0.31 ± 1.3	-0.77 ± 0.95	**0.03**
	LV EF (%)	66.69 ± 5.92	72.05 ± 6.09	**<0.0001**
	Septum (z-score)	+2.18 ± 1.4	+0.61 ± 0.91	**<0.0001**
	LV posterior wall (z-score)	+1.74 ± 1.2	+0.4 ± 0.9	**<0.0001**
	LV mass index (g/m^2,7^)	41.74 (17.72 – 108.39)	32.57 (19.07 – 74.7)	**0.0010**
	Frequency of individuals with LV mass index > P95	24 (43.63%)	0 (0%)	**<0.0001**
**Relative wall thickness (RWT)**		**0.44 ± 0.08**	**0.36 ± 0.05**	**<0.0001**
	Frequency of individuals with RWT > 0.42	35 (63.63%)	0 (0%)	**<0.0001**
	LA diameter (z-score)	-0.29 ± 0.85	-0.16 ± 1	0.45
	LA volume (ml/m^2^)	15.63 ± 05.08	16.19 ± 04.54	0.5385
	Mitral E (cm/s)	92.79 ± 21.47	102.91± 17.41	**0.0077**
	Mitral A (cm/s)	61.6 (27.6 – 142)	51.40 (33.8 – 93.6)	**0.0180**
	Mitral E/A (cm/s)	1.57 ± 0.56	1.96 ± 0.51	**0.0003**
	Tissue Doppler septal e’ (cm/s)	10.7 ± 2.61	13.46 ± 2.2	**<0.0001**
	Tissue Doppler lateral e’ (cm/s)	14.2 (6.58 – 29.2)	18.8 (11.5 – 33.1)	**<0.0001**
	E/e’ (cm/s)	6.99 (4.75 – 14.2)	6.38 (3.88 – 11.11)	**0.0092**
**2DST Echocardiographic parameters**	
	Left atrial longitudinal reservoir strain (%)	48.22 ± 10.62	58.52 ± 10.7	**<0.0001**
	Left atrial longitudinal conduit strain (%)	37.26 ± 09.77	43.79 ± 10.13	**0.0008**
	Left atrial longitudinal contractile strain (%)	11.8 (1.60 – 19.6)	14.30 (5.20 – 27.2)	**0.0009**
	Left atrial stiffness index (%^-1^)	0.14 (0.08 – 0.48)	0.11 (0.06 – 0.23)	**<0.0001**
	Left atrial filling index (cm/s x %^-1^)	2.02 ± 0.63	1.8 ± 0.39	**0.0335**
	Left ventricular peak systolic global longitudinal strain (%)	19.4 (9 – 36.4)	21.9 (18.1 – 27.2)	<0.0001

RVDD: right ventricle diastolic diameter; LVDD: left ventricular diastolic diameter; LVSD: left ventricular systolic diameter; LV: left ventricle; LA: left atrium; P95: 95^th^percentile; RWT: relative wall thickness; bold indicates p<0.05; continuous data are presented as mean ± standard-deviation or median (minimum – maximum) and categorical data as frequency and percentage.

The underlying causes of CKD were congenital anomalies of kidney and urinary tract (CAKUT) in 34 (61.8%), tubulopathies in seven (12.7%), glomerulopathies in six (11%) and miscellanea in eight (14.5%) patients. The median duration of the disease was 8.1 (0.83 - 17.5) years. There were seven (12.8%) CKD stage I, 4 (7.3%) CKD stage II, 12 (21.8%) CKD stage III, two (3.6%) CKD stage IV and 30 (54.5%) CKD stage V patients.

Nineteen (34.5%) patients did not have hypertension; 21 (38.2%) had controlled hypertension (systolic and diastolic blood pressure ≤ 95^th^percentile, under treatment) and 15 (27.3%) had uncontrolled hypertension (systolic and/or diastolic blood pressure > 95^th^percentile, despite treatment). Antihypertensive drugs included amlodipine (25.5%), enalapril (14.5%), carvedilol (9%), losartan (7.3%), atenolol (3.6%), hydralazine (3.6%) and furosemide (3.6%). Of patients treating hypertension, 68% received a single agent, 16% two agents and 16% three agents. The median value of hematocrit was 35.6% (27.2% - 46.9%), of serum phosphorus 4.5mg/dL (2.4mg/dl - 7.2mg/dL) and of PTH 128pg/mL (13 pg/mL - 628 pg/mL). 26 (47.3%) CKD patients had anemia,^14^20 (36.4%) had phosphorus levels above the expected threshold^15^and 33 (60%) showed PTH levels above target values.^15^

Among the 30 dialysis patients, 14 (46.7%) were on hemodialysis and 16 (53.3%) on peritoneal dialysis. The average duration of dialysis was 2.25 ± 1.2 years in the hemodialysis group and 1.35 ± 1.09 years in the peritoneal dialysis group.

### Standard echocardiogram: CKD patients vs. controls

LVEF was normal (> 55%), in all individuals although lower in patients than in controls. LVMI was higher among CKD patients. Both LA diameter and volume were similar between the two groups. Even though average E/e’ was higher in CKD patients, it was above normal limits in only one individual (E/e’ = 14.2)^23^( Table 1 ). Among CKD patients, 14 (25.4%) showed normal ventricular geometry, 17 (30.9%) concentric remodeling, 18 (32.7%) concentric hypertrophy and 6 (11%) eccentric hypertrophy.

### 2DST echocardiogram: CKD patients vs. controls

Satisfactory images were obtained from all CKD patients and controls; no individuals were excluded from myocardial strain evaluation. Patients showed lower values of all LA strain components (reservoir, conduit and contraction), higher LA stiffness and filling index and lower LV peak systolic global longitudinal strain ( Table 1 ).

### LA strain vs. conventional echocardiographic parameters in CKD patients

In the CKD group, LA reservoir strain correlated negatively with LV mass index and E/e’. LA reservoir strain correlated positively with lateral e’, septal e’ and average e’. LA conduit strain correlated negatively with LV mass index and E/e’. LA conduit strain correlated positively with lateral e’, septal e’ and average e’. LA contractile strain correlated negatively with mitral E and E/e’. LA stiffness index correlated negatively with lateral e’, septal e’ and average e’. LA stiffness index correlated positively with LV mass index, mitral E, mitral A and E/e’. LA filling index correlated positively with LV mass index, mitral E and E/e’ ( Table 2 ).

**Table 2:** Correlations between conventional echocardiographic parameters and left atrium strain components, stiffness index and filling index in the chronic kidney disease group

	Left atrium longitudinal reservoir strain (%)	Left atrium longitudinal conduit strain (%)	Left atrium longitudinal contractile strain (%)	Left atrium stiffness index	Left atrium filling index
LVEF (%)	0.12 (0.3748)	0.12 (0.4024)	0.01 (0.9272)	-0.10 (0.4496)	0.01 (0.9241)
LVMI (g/m^2,7^)	-0.48 (0.0002)	**-0.42 (0.0016)**	-0.18 (0.1994)	0.50 (0.0001)	**0.37 (0.0059)**
RWT	-0.13 (0.3263)	-0.10 (0.4680)	-0.15 (0.2861)	0.11 (0.4391)	0.13 (0.3388)
LA volume (mm/m^2^)	-0.03 (0.8013)	-0.11 (0.4357)	0.10 (0.4686)	-0.04 (0.7459)	0.10 (0.4507)
Mitral E (cm/s)	-0.10 (0.4659)	0.11 (0.4355)	**-0.28 (0.0370)**	**0.35 (0.0082)**	**0.70 (<0.0001)**
Mitral A (cm/s)	-0.14 (0.3204)	-0.12 (0.3945)	0.00 (0.9860)	**0.29 (0.0347)**	0.25 (0.0668)
Mitral E/A (cm/s)	0.11 (0.4307)	0.21 (0.1225)	-0.15 (0.2765)	-0.11 (0.4359)	0.16 (0.2354)
Tissue Doppler lateral e’ (cm/s)	**0.46 (0.0004)**	**0.46 (0.0004)**	0.10 (0.4573)	**-0.57 (<0.0001)**	-0.21 (0.1328)
Tissue Doppler septal e’ (cm/s)	**0.34 (0.0106)**	**0.40 (0.0027)**	-0.02 (0.8863)	**-0.32 (0.0185)**	0.13 (0.3585)
Average e’ (cm/s)	**0.49 (0.0001)**	**0.50 (0.0001)**	0.08 (0.5658)	**-0.56 (<0.0001)**	-0.09 (0.4922)
E/e’ (cm/s)	-0.48 (0.0002)	-0.30 (0.0251)	-0.33 (0.0142)	0.83 (<0.0001)	**0.73 (<0.0001)**

LVEF: left ventricular ejection fraction; LVMI: left ventricular mass index; RWT: relative wall thickness; LV: left ventricle; LA: left atrium; data are presented as Spearman rank coefficient of correlation (p-value); bold indicates p<0.05

### LA strain vs. LV peak systolic global longitudinal strain in CKD patients

LV peak systolic global longitudinal strain correlated positively with LA reservoir strain and LA conduit *strain* and showed a negative correlation with LA stiffness index ( Figure 2 ).

**Figure 2:** Correlations between left ventricular peak systolic global longitudinal strain and left atrial strain parameters. LV: left ventricle; LA: left atrium.

### 2DST echocardiographic parameters according to CKD stage

CKD stage showed weak negative correlation with LA reservoir strain and conduit strain. A moderate positive correlation was detected between CKD stage and LA stiffness index ( Figure 3 ). LA contractile strain, LA filling index and LV peak systolic global longitudinal strain did not correlate with CKD stage.

**Figure 3:** Left atrium (LA) strain parameters according to chronic kidney disease (CKD) stage.

### 2DST echocardiographic parameters according to LV mass index in CKD

Patients with CKD and LV mass index > 95^th^percentile (P95) showed lower values of LA reservoir and conduit strain, and higher stiffness index and filling index. LA contractile strain and LV peak systolic global longitudinal strain were similar between groups ( Table 3 ).

**Table 3:** Two-dimensional speckle tracking (2DST) echocardiography: CKD patients with LV hypertrophy vs. CKD patients without LV hypertrophy

2DST echocardiogram	LV mass index ≤ P95 (n=31)	LV mass index > P95 (n=24)	p-value
Left atrial longitudinal reservoir strain (%)	52,99 ± 9,52	42,05 ± 8,74	<0,0001
Left atrial longitudinal conduit strain (%)	41 ± 9,63	32,43 ± 7,74	0,0005
Left atrial longitudinal contractile strain (%)	12,5 (4,2 – 19,6)	9,3 (1,6 – 16,2)	0,1144
Left atrial stiffness index (%^-1^)	0,13 (0,08 – 0,23)	0,23 (0,11 – 0,48)	<0,0001
Left atrial filling index (cm/s x %^-1^)	1,74 ± 0,47	2,39 ± 0,63	0,0001
Left ventricular peak systolic global longitudinal strain (%)	19,4 (15,2 – 36,4)	19,35 (9 – 27,5)	0,4152

P95: 95^th^percentile. Bold indicates p<0.05; continuous data are presented as mean ± standard-deviation or median (minimum – maximum).

### 2DST echocardiographic parameters according to LV geometry in CKD

LV peak systolic global longitudinal strain was similar in the four LV geometry groups.

Comparing patients with concentric hypertrophy and patients with normal LV geometry, the former group showed lower LA reservoir strain (40.97 ± 9.53% *vs* . 54.36 ± 8.6%) and conduit strain (32.23 ± 8.59% *vs* . 41.89 ± 9.32%), and higher LA stiffness index [0.23 (0.11–0.48) %^-1^ *vs* . 0.12 (0.08–0.23) %^-1^] and LA filling index (2.43 ± 0.59 cm/s x %^-1^ *vs* . 1.73 ± 0.49 cm/s x %^-1^) (p<0.05).

Similarly, comparing patients with concentric hypertrophy and patients with concentric remodeling, the former group showed lower reservoir strain (40.97 ± 9.53% *vs* . 51.86 ± 10.34%), and higher LA stiffness index [0.23 (0.11 – 0.48) %^-1^ *vs.* 0.13 (0.08 – 0.19) %^-1^] and LA filling index (2.43 ± 0.59 cm/s x %^-1^vs. 1.74±0.46 cm/s x %^-1^) (p<0.05).

There were no significant differences between LA strain parameters comparing patients with LV concentric and eccentric hypertrophy.

### 2DST echocardiographic parameters according to blood pressure control in CKD

CKD patients with uncontrolled hypertension showed lower LA longitudinal reservoir and conduit strain. LV peak systolic global longitudinal strain was also reduced in the group with uncontrolled hypertension ( Table 4 ).

**Table 4:** 2DST echocardiogram: CKD patients with normal pressure/controlled hypertension vs. CKD patients with uncontrolled hypertension

No hypertension/controlled hypertension (n=40)	2DST echocardiogram	Uncontrolled hypertension (n=15)	p-value
Left atrial longitudinal reservoir strain (%)	50,6 ± 9,7	41,9 ± 10,6	0,0055
Left atrial longitudinal conduit strain (%)	38,30 (22,3 – 63,60)	32,00 (11,60 – 48,20)	0,0190
Left atrial longitudinal contractile strain (%)	12,10 (1,60 – 19,40)	10,00 (3,00 – 19,60)	0,3304
Left atrial stiffness index (%^-1^)	0,14 (0,08 – 0,28)	0,15 (0,10 – 0,48)	0,1695
Left atrial filling index (cm/s x %^-1^)	1,93 ± 0,57	2,27 ± 0,72	0,1093
Left ventricular peak systolic global longitudinal strain (%)	19,85 (15,20 – 36,40)	18,80 (9,00 – 24,20)	0,0482

Bold indicates p<0.05; continuous data are presented as mean ± standard-deviation or median (minimum – maximum).

### Comparisons between non-dialysis and dialysis CKD patients

Non-dialysis and dialysis CKD patients were similar regarding age and gender distribution. Dialysis patients had lower dry weight, height and body surface area.

Standard and 2DST echocardiographic parameters of non-dialysis and dialysis patients are presented in Table 5 . LVMI and E/e’ were higher among dialysis patients, whereas LVEF and LA volume were similar between groups. There was a significant association between abnormal LV geometry and dialysis. Only one in five patients with eccentric hypertrophy was not under dialysis. Despite that, the association between eccentric hypertrophy and dialysis was not significant, probably due to the small number of patients in our sample (p=0.20). LA reservoir strain was lower, and LA stiffness index was higher in the dialysis group.

**Table 5:** Non-dialysis vs. dialysis CKD patients: demographic data, standard and 2DST echocardiographic parameters

Demographic data		Non-Dialysis (n=25)	Dialysis (n=30)	p-value
Age (years)		10.6 ± 4.1	8.5 ± 5	0.1100
Gender (male)		13 (52.00%)	23 (76.67%)	0.1029
Dry Weight (kg)		31.00 (14.50 – 100.00)	18.05 (05.00 – 73.00)	0.0075
Height (m)		01.04 (01.00 – 01.85)	01.11 (00.66 – 01.67)	**0.0026**
BSA (m^2^)		01.12 ± 00.39	00.85 ± 00.41	**0.0046**
Heart rate (bpm)		100 ± 13	105 ± 14	**0.1800**
**Standard echocardiographic parameters**				
	LVDD (z-score)	-0.78 ± 1.13	-0.11 ± 1.35	0.0540
	LVSD (z-score)	-0.62 ± 1.14	-0.05 ± 1.37	0.1070
	LV EF (%)	66.35 ± 6.48	66.97 ± 5.51	0.7082
	Septum (z-score)	+1.74 ± 1.21	+2.5 ± 1.48	**0.0363**
	LV posterior wall (z-score)	+1.30 ± 0.97	+2.1 ± 1.27	**0.0127**
	LV mass index (g/m^2,7^)	32.37 (17.72 – 54.1)	51.6 (21.58 – 108.39)	**<0.0001**
	Frequency of individuals with LV mass index > P95	6 (24.00%)	18 (60.00%)	**0.0160**
	RWT	0.43 (0.31 – 0.64)	0.46 (0.25 – 0.63)	0.2452
	Frequency of individuals with RWT > 0.42	14 (56.00%)	21 (70.00%)	0.4276
	Frequency of abnormal LV geometry	15 (60%)	26 (86.6%)	**0.032**
	LA diameter (z-score)	-0.19 ± 0.91	-0.38 ± 0.81	0.3974
	LA volume (ml/m^2^)	16.18 (9.15 – 28.57)	13 (9.13 – 24)	0.1369
	Mitral E (cm/s)	92.98 ±16.51	92.64 ± 25.16	0.9524
	Mitral A (cm/s)	55.1 (27.6 – 102)	64.45 (36.9 – 142)	**0.0006**
	Mitral E/A (cm/s)	1.87 ± 0.59	1.33 ± 0.41	**0.0003**
	Tissue Doppler septal e’ (cm/s)	11.99 ± 2.25	9.63 ± 2.42	**0.0004**
	Tissue Doppler lateral e’ (cm/s)	16.66 ± 3.61	13.11 ± 3.58	**0.0006**
	E/e’ (cm/s)	6.3 (4.75 – 9.54)	7.89 (5.47 – 14.2)	**0.0007**
**2DST echocardiographic parameters**				
	Left atrial longitudinal reservoir strain (%)	52.24 ± 9.58	44.87 ± 10.42	**0.0086**
	Left atrial longitudinal conduit strain (%)	40.0 2 ± 9.82	34.96 ± 9.26	0.0563
	Left atrial longitudinal contractile strain (%)	12.2 (4.2 – 19.6)	9.8 (1.6 – 17.5)	0.1281
	Left atrial stiffness index (%^-1^)	0.13 ± 0.05	0.20 ± 0.08	**0.0005**
	Left atrial filling index (cm/s x %^-1^)	1.86 ± 0.55	2.15 ± 0.66	0.0766
	Left ventricular peak systolic global longitudinal strain (%)	19.4 (16.1 – 26.9)	19.35 (9 – 36.4)	0.7738

RVDD: right ventricle diastolic diameter; LVDD: left ventricular diastolic diameter; LVSD: left ventricular systolic diameter; LV: left ventricle; LA: left atrium; P95: 95th percentile; RWT: relative wall thickness; Bold indicates p<0.05; continuous data are presented as mean ± standard-deviation or median (minimum – maximum).

### Comparisons between patients on peritoneal dialysis and hemodialysis

LA strain parameters were not different comparing peritoneal and hemodialysis patients ( Table 6 ).

**Table 6:** Peritoneal vs. hemodialysis: speckle-tracking parameters

Strain parameters	Peritoneal (n=14)	Hemodialysis (n=16)	p-value
Left ventricular peak systolic global longitudinal strain (%)	21.20±6.47	19.55±2.83	0.3894
Left atrial longitudinal reservoir strain (%)	41.36±10.41	47.94±9.72	0.0859
Left atrial longitudinal conduit strain (%)	31.63±9.24	37.88±8.52	0.0661
Left atrial longitudinal contractile strain (%)	8.40 (4.50 – 16.00)	12.15 (1.60 – 17.50)	0.9834
Left atrial stiffness index (%^-1^)	0.21 (0.11 – 0.48)	0.18 (0.10 – 0.28)	0.3816
Left atrial filling index (cm/s x %^-1^)	2.29 (1.25 – 2.95)	2.14 (0.88 – 3.33)	0.6100

### Intra and inter-observer variability

Adequate ICC (> 0.80) was obtained for all 2DST echocardiographic parameters for intra and inter-observer variability, except for LA contractile strain (ICC = 0.61 for inter-observer variability) ( Table 7 ). The main study results are pointed out in the Central Illustration .

**Table 7:** Intra and inter-observer variability of speckle-tracking parameters

Parameters	Intra-observer test		Inter-observer test	
	ICC (CI)	p-value	ICC (CI)	p-value
Left atrial longitudinal reservoir strain (%)	0.99 (0.98 – 1.00)	<0.0001	0.83 (0.57 – 0.93)	<0.0001
Left atrial longitudinal conduit strain (%)	0.99 (0.98 – 1.00)	<0.0001	0.87 (0.67 – 0.95)	<0.0001
Left atrial longitudinal contractile strain (%)	0.92 (0.81 – 0.97)	<0.0001	0.61 (0.03 – 0.84)	<0.0001
Left ventricular peak systolic global longitudinal strain (%)	0.98 (0.94 – 0.99)	<0.0001	0.89 (0.74 – 0.96)	<0.0001

ICC: intraclass correlation coefficient.

## Discussion

This study stands out for the detection of subclinical impaired LA strain in pediatric CKD patients at different stages of the disease, with great feasibility and reproducibility. It was also possible to demonstrate significant associations between LA strain impairment and previously demonstrated cardiovascular risk factors in the CKD population, like LV hypertrophy and uncontrolled systemic arterial hypertension.

Previous works using tissue Doppler imaging suggested impaired LV diastolic parameters early in the progression of CKD, with the worst values being recorded in patients undergoing maintenance dialysis.^24^Nevertheless, only one CKD patient in our study showed average E/e’ greater than 14, one of the key noninvasive markers of diastolic dysfunction among patients with preserved ejection fraction, according to ASE adult guidelines.^23^

There is growing evidence that current algorithms for evaluation of diastolic dysfunction in adults are not as reliable in pediatric populations. Moreover, in children with various types of cardiomyopathies, criteria for diastolic dysfunction were discrepant in most patients and half of them exhibited E/e’ values within the normal range for age.^5^In line with the study of Morris et al.^25^our data favors LA reservoir strain and LA stiffness index as additive diastolic parameters, with prognostic value yet to be proven among pediatric patients.^25^

Despite significant reduction of LA reservoir strain, LA volume in our pediatric CKD and control groups were alike. Indeed, LA reservoir strain alterations had been recently shown to precede LA volume increase, classically known as a hallmark of diastolic dysfunction.^26^Studies in adult CKD patients have depicted an inverse correlation between LA strain and mean pulmonary capillary wedge pressure obtained by catheterization, independently of LA volume.^27^Nakanishi et al.^28^hypothesized different underlying mechanisms that may be implicated in LA dysfunction in CKD, with still normal LA volume: chronic inflammatory state, LA myocardium fibrosis induced by chronic renin–angiotensin–aldosterone system activation, sympathetic stimulation and oxidative stress.^28^

There are scant published data regarding diastolic function of pediatric CKD patients through the different stages of the disease. In our study, CKD stage correlated negatively with LA reservoir strain and positively with LA stiffness index, suggesting that these novel parameters may reflect kidney disease progression and diastolic function deterioration, even in the absence of overt heart failure. Indeed, Gan et al.^29^demonstrated the prognostic value of LA reservoir strain as an independent predictor of progression of renal dysfunction in stage 3/4 adult CKD patients, without previous cardiac history and stable renal function.^29^

Although our CKD patients with and without LVMI > 95^th^percentile showed similar LV systolic strain, LA strain impairment was significantly associated with LV hypertrophy. Moreover, CKD patients with concentric hypertrophy had lower LA reservoir strain, and higher LA stiffness index and LA filling index than CKD patients with normal LV geometry or concentric remodeling. This information seems clinically relevant, since LV hypertrophy is the most important indicator of cardiovascular risk in CKD population and abnormal patterns of LV geometry adversely affect prognosis.^30 – 32^

Uncontrolled hypertension in our CKD patients was frequent (27.3%) and associated with lower LA reservoir strain and higher LA stiffness index. These findings may impact on prognosis, since a recent study from Zhao et al.^33^demonstrated that LA stiffness index precedes LV hypertrophy, besides being independently correlated with individual target organ damage in adult patients with hypertension.

Traditionally, both systolic and diastolic functions are evaluated as separate phases. However, they are closely interrelated through several mechanisms, such as the Frank–Starling mechanism, wherein enhanced filling increases contractility, which in turn increases elastic recoil in early diastole. Corroborating previous studies that have described systolic and diastolic coupling, we have documented in our pediatric CKD group significant correlation between LV peak systolic global longitudinal strain and LA reservoir strain, conduit strain and stiffness index.^34^

LA reservoir strain was lower, and LA stiffness index was higher among our CKD patients under dialysis, compared to non-dialysis patients. The impact of dialysis on diastolic function was also investigated by Doan et al.^35^who evaluated LA strain prior to, during and after hemodialysis sessions. The authors described significant reduction of LA strain in mid-dialysis, with return to baseline values post-dialysis.^35^

### Study limitations

Possible limitations include the small number of patients enrolled and the single center nature of the study, which may preclude generalizations of conclusions to larger populations. Since we are a pediatric nephrology referral center, the high prevalence of end-stage renal disease among our sample (54% in stage V) may have contributed to worse LA deformation.

Standard and 2DST echocardiograms were analyzed by the same pediatric cardiologist, blinded to medical records. This examiner was, however, aware of the subjects as either patients or controls, since children under dialysis usually carry a catheter (peritoneal or central venous). Nevertheless, the second observer was absolutely blinded for the group allocation and ICC was considered adequate.

Patients undergoing dialysis were examined closer to their clinically estimated dry weight, since we did not assess blood volume.

We did not include serum levels of pro-Brain Natriuretic Peptide (pro-BNP) or inflammation mediators in the present study, since they are not routinely ordered by the physicians at our outpatients’ clinics. Moreover, we did not investigate possible correlations between LA strain and exercise capacity of our pediatric CKD patients. All that could have helped to detect subtle myocardial impairment associated with LA strain compromise.

Although hemodialysis is usually associated with greater cardiovascular compromise than peritoneal dialysis,^33^we did not find significant difference of LA strain parameters between these two types of renal replacement therapy, perhaps due to the small sample size in each group of patients.

Since our study was meant to be a cross-sectional one, prognostic implications of LA strain evaluation in pediatric CKD patients, including morbidity and mortality, were not investigated.

## Conclusion

LA strain evaluation proved to be a feasible tool concerning diastolic evaluation in a pediatric CKD population. The present study documented significant associations between LA strain impairment and cardiovascular risk factors in this population. Since diastolic dysfunction has a strong prognostic value in CKD, incorporation of LA strain in routine echocardiographic evaluation of this pediatric population seems to be an appropriate strategy.

Longitudinal assessment using these novel non-invasive indices may unfold the effects of CKD on long-term cardiovascular health throughout children development.
